# Top predator restricts the niche breadth of prey: effects of assisted colonization of Tasmanian devils on a widespread omnivorous prey

**DOI:** 10.1098/rspb.2022.2113

**Published:** 2023-03-29

**Authors:** Vincent P. Scoleri, Janeane Ingram, Christopher N. Johnson, Menna E. Jones

**Affiliations:** ^1^ School of Natural Sciences, University of Tasmania, Sandy Bay 7005, Australia; ^2^ School of Geography, Planning and Spatial Sciences, University of Tasmania, Sandy Bay 7005, Australia; ^3^ Australian Research Council Centre of Excellence for Australian Biodiversity and Heritage, University of Tasmania, Sandy Bay 7005, Australia

**Keywords:** biological control, native predator introduction, predator–prey, ecological restoration

## Abstract

Few landscape-scale experiments test the effects of predators on the abundance and distribution of prey across habitat gradients. We use the assisted colonization of a top predator, the Tasmanian devil (*Sarcophilus harrisii*), to test the impacts of predation on the abundance, habitat use and temporal activity of a widespread prey species, the omnivorous common brushtail possum (*Trichosurus vulpecula*). Before introduction of devils to Maria Island, Tasmania, Australia, in 2012, possums were abundant in open grasslands as well as forests. Predation by devils caused high mortality of possums in grasslands, but individuals with access to trees had a higher survival probability. Possum abundance declined across the whole island from 2012–2016, as possums disappeared almost completely from grasslands and declined in drier forests with more open understorey. Abundance remained stable in wet forests, which are not preferred habitat for possums but provide better refuge from devils. Abundance and habitat use of possums remained unchanged at a control site on the adjacent Tasmanian mainland, where the devil population was low and stable. This study demonstrates how spatial variation in predator-caused mortality can limit both abundance and habitat breadth in generalist prey species, excluding them entirely from certain habitats.

## 1. Introduction

Top predators can limit populations of prey by increasing mortality and reducing abundance, and by causing changes in prey distribution, including shifts in habitat use, in response to risk and the perceived risk of predation [1]. The loss of top predators can therefore result in prey species increasing in both local abundance and habitat breadth [2]. Conversely, restoration of top predators may be a powerful tool for managing populations of prey species and assist in the recovery of degraded ecosystems [3].

Our understanding of the potential benefits from restoring top predators is strongly influenced by well-studied examples that provide only correlative evidence [4] such as the interpretation of historical changes in predator and prey populations [5]. In such cases, it can be difficult to identify the mechanisms of change, and it may not be possible to exclude potentially confounding ecological changes due to the long timescales involved. The reintroduction of grey wolves (*Canis lupus*) to the Greater Yellowstone ecosystem, for example, was followed by the decreasing abundance and habitat breadth of elk (*Cervus canadensis*) with cascading effects on other species [1]. Because these changes unfolded over several decades, direct study of predator–prey interactions was limited, and, the mechanisms by which wolves affected the ecology of elk—whether through the direct demographic effects of predation, shifts in distribution, or the influence of bottom-up effects—are still being debated [6–8].

Other examples of top predator restoration occur in regions with strong human influences on both predator and prey species [9]. For example, large terrestrial predators are re-colonizing western Europe [10], in landscapes that are largely human dominated. The long-term effects of top predator restoration and re-colonization are often masked by human control of wildlife populations and potential trophic interactions [11]. For these reasons, studies of the effects from recovering and re-colonizing top predator populations rarely occur without human influence on either the predator or prey populations. Here, we investigate the effects from the assisted colonization of a native predator on a widespread prey species in isolation from human influence.

The Tasmanian devil (*Sarcophilus harrisii*; hereafter ‘devil’) is the largest extant marsupial carnivore and the top terrestrial predator on the island of Tasmania, Australia [12]. Devils have declined on the Tasmanian mainland since the mid-1990s due to a novel transmissible cancer, devil facial tumour disease (DFTD) [13]. In response to this threat, a founder group of 28 disease-free devils was translocated to Maria Island off the east coast of Tasmania in 2012–2013 to establish a wild-living insurance population [14,15]. Over the subsequent 4 years the devil population rapidly grew to approximately 100 individuals, reaching the predicted carrying capacity for the island [16]. Maria Island provides the range of habitats occupied by devils on mainland Tasmania and a full complement of prey species, including marsupial herbivores introduced to the island from the 1950s to the 1970s that are now widespread and abundant [17]. Maria island has been a National Park since 1972 with no permanent residents and minimal human influence.

The common brushtail possum (*Trichosurus vulpecula*; hereafter ‘possum’) is a major prey species of the devil on mainland Tasmania [18]. Possums are typically forest-dwelling arboreal folivores that also forage on the ground to varying extents [19]. Possums can expand their diet by opportunistic predation of birds and other small vertebrates [20], and by scavenging carrion [21]. Prior to the assisted colonization of devils, possums were widespread on Maria Island and occupied all habitat types including grasslands where they were mostly ground dwelling. In some coastal areas, individual possums preyed on nesting seabirds and denned in wombat burrows [21].

In this study, we investigated the effects from the increasing devil population on the abundance and distribution of possums on Maria Island. We predicted that possums would be at greatest risk from devil predation in grassland habitats with limited tree cover to escape from a terrestrial predator, and that the possum population would contract to forested habitats to reduce predation risk from devils. We deployed GPS collars to track the movements (and individual survival) of possums in a mixed grassland/forest landscape. In addition, camera surveys and spotlight transect surveys using distance sampling techniques were used to measure changes in possum abundance, distribution and temporal patterns of activity across the whole island. Camera surveys were also conducted at a control site on the adjacent Tasmanian mainland where devils remained at low density throughout the study. The study was conducted from 2010 and continued through the years of devil introduction (in 2012–2013) on Maria Island to 2019.

## 2. Methods

### (a) Study sites

**Figure 1.**
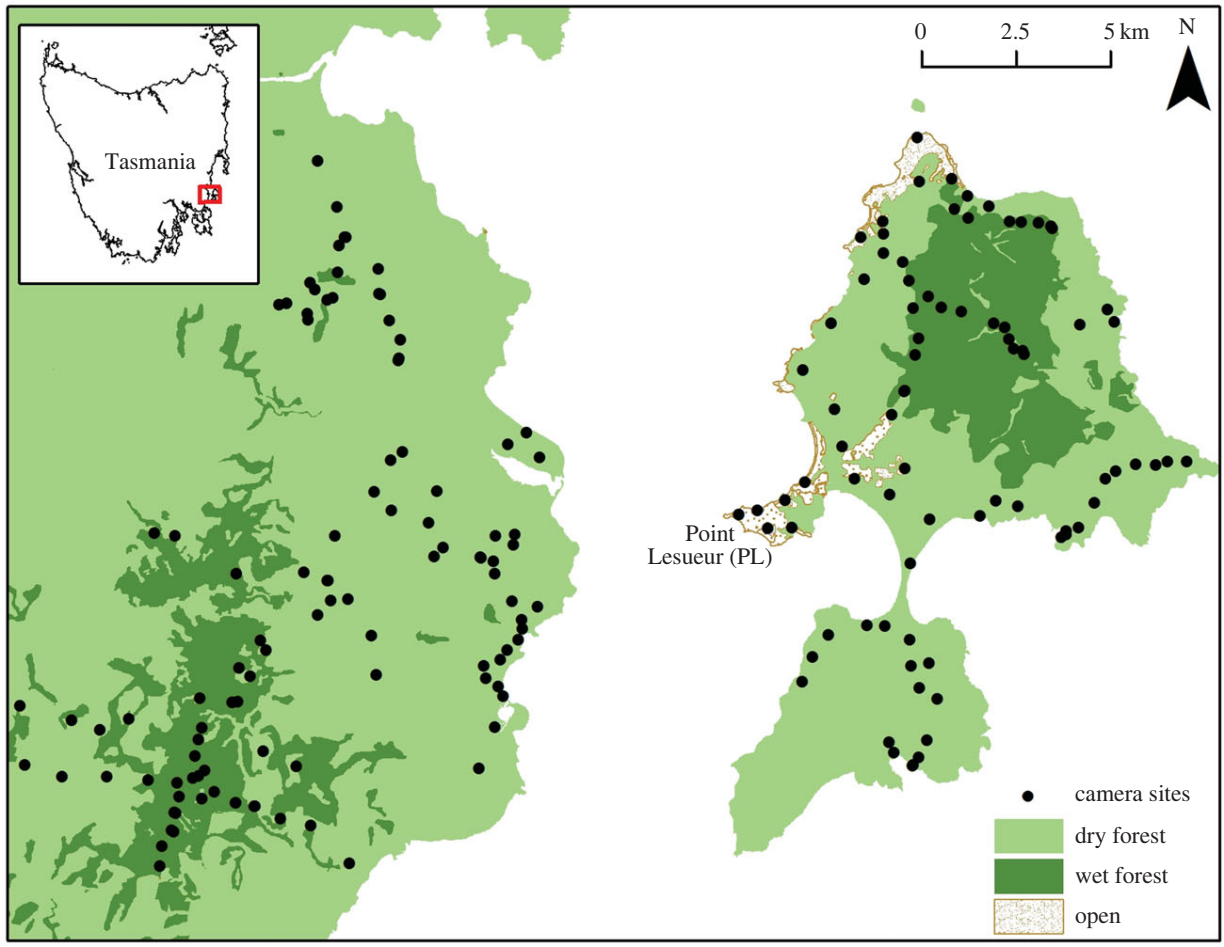
Location of study sites on the east coast of Tasmania, Australia (inset). Devils were introduced to Maria Island in 2012–2013. The control site was located on the adjacent mainland where a low and stable devil population occurs naturally. Camera traps were distributed in both wet and dry forests, and grassland habitats. Possum densities were monitored in grassland habitats (approx. 1000 ha) on Maria Island by spotlight surveys from 2010 to 2018. Individual possums were fitted with GPS collars at Point Lesueur on Maria Island from 2014 to 2015.

Maria Island is an approximately 100 km^2^ island 4 km off the east coast of Tasmania, Australia (figure 1). The island is mostly covered by dry forest dominated by *Eucalyptus globulus*, *E. obliqua*, *E. pulchella* and *E. amygdalina*, with some *Allocasuarina verticillata*. Dry forest as defined includes limited areas of coastal dry heath and sand dunes. At higher altitudes (up to 700 m) dry forests are replaced by wet forests, which are denser and more structurally complex, with several distinct habitat types, including: tall woodland on talus; plateau-shelf tall open forest, tall woodland with wet sclerophyll understorey; and mountain-top heath [22] (figure 1). Before protection of the island as National Park, native vegetation was cleared on the coastal flats creating open grasslands over approximately 5.5% of the island [22]. The grasslands are maintained as closely cropped ‘marsupial lawns’ by common bare-nosed wombats (*Vombatus ursinus*), forester kangaroos *(Macropus giganteus)*, Bennett's wallabies (*M. rufogriseus rufogriseus*), Tasmanian pademelons (*Thylogale billardierii*) and possums [23].

The control site had similar vegetation and climate to Maria Island and was located on the adjacent Tasmanian mainland near Rheban (figure 1). The devil population at this site was low density and stable, having been affected by DFTD since 2003 [24].

### (b) Survival of individual possums in relation to habitat use

**Figure 2.**
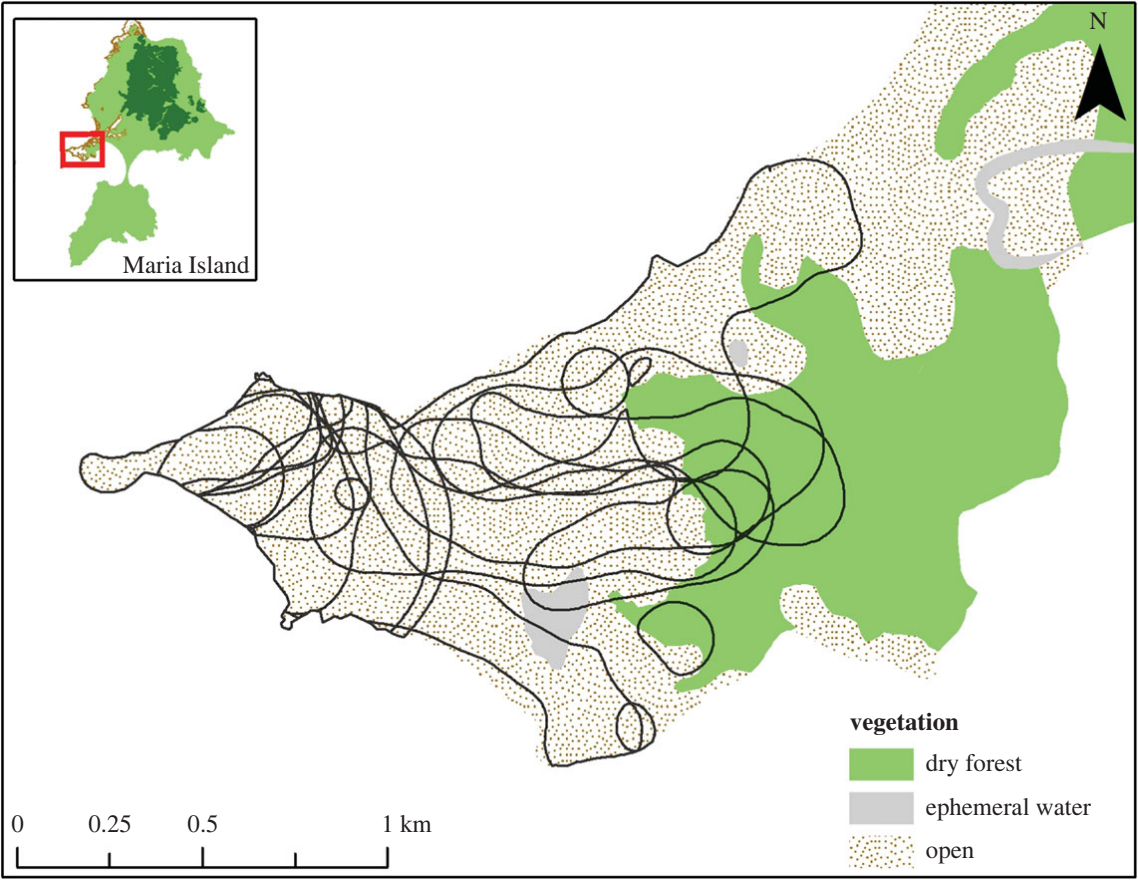
HR areas calculated for 18 GPS-collared possums within open grassland and dry forest habitats with access to trees at Point Lesueur, on Maria Island (inset) from March 2014 to June 2015.

Possums living in mixed grassland and dry forest habitats at Point Lesueur, the western-most part of Maria Island (figure 2), were fitted with GPS collars (Sirtrack, Havelock, New Zealand) between February 2014 and March 2015. Possums were captured in PVC pipe traps, modified cage traps, or by spotlighting and hand-netting at night. GPS collars were set to record a satellite fix every 15 min, from 18.00 h to 06.00 h AEST 4 days per week. Possums were re-located by radiotracking the VHF transmitter in the collar and their fate was recorded as either alive, killed or unknown. Surviving possums were recaptured for the removal of collars. Evidence that collared possums had been killed or eaten by a devil consisted of blood, fur, tracks, drag marks and damage to the GPS collar at a kill site. The category ‘unknown’ was assigned to possums where the GPS collar was found but there was no clear evidence of predation by devils.

Data from GPS collars were analysed for 18 possums (12 males, 6 females) in total (figure 2). A further nine possums were collared but provided too few fixes for estimation of home ranges (HRs). Collars were downloaded using *Sirtrack* GPS software (v. 1.5.3). Geographical coordinates from each possum were exported as separate .csv files and projected onto the Maria Island GIS vegetation layer in TasVeg v. 3.0 [25](Department of Primary Industries, Parks, Water and Environment, 2013) with files uploaded to ArcGIS v. 10.2 [26]. Fixes that appeared spurious (e.g. in the ocean), or that took longer than 90 s to obtain, were removed and the cleaned data compiled for all collared possums.

We estimated the core HR for each possum using the biased random bridge (BRB) kernel method [27,28] in the R package *adehabitatHR* version 0.4.15 [29]. The BRB estimates HR by placing kernel functions over each step (track) travelled by the animal between consecutive GPS fixes, rather than over the fixes themselves, resulting in a more realistic estimate of the animals' actual movements. Parameters were set as follows: (i) the maximum amount of time (in seconds) allowed for steps built by successive relocations was TMax = 1000 s, based on the 15 min (i.e. 1000 s) fix schedule for GPS collars, and (ii) the smallest distance for a possum recorded as not moving (Lmin) was set at 20 m to account for error within the GPS collar fixes. Defining intensive small-scale movements or resting by possums (hmin) was set to 50 m.

Home-range polygons for individual possums were overlayed on a vegetation shapefile of Maria Island (TasVeg v. 3.0; figure 2). Vegetation codes were categorized as either ‘grassland’ or ‘forest’ habitats. The area of each possum's HR within each habitat type was calculated (electronic supplementary material, table S1). A survival analysis was performed to investigate the survival (killed, alive or unknown) of GPS-collared possums in relation to whether they had forest within their HR. Survival probability estimates of possums whose HRs included some areas of forest versus those that did not were compared using a log-rank test of these two estimates over time. Due to the relatively low sample size of GPS-collared possums and the number confirmed as killed (*n* = 13) we did not investigate other variables that might predict survival.

### (c) Change in abundance of possums in open grassland habitat

Possum abundances in grassland were estimated from standardized spotlight survey counts conducted by Parks and Wildlife Service field staff on Maria Island in autumn (May) and winter (August) from 2010 to 2018 (i.e. 16 surveys in each season). Three survey counts were completed over a one-week period in each season on non-consecutive nights. A vehicle, with a driver, observer and recorder was driven at slow speed (approx. 10 km hr^−1^) along 15 transects located on straight sections of gravel roads, tracks and grasslands at Darlington, Return Point and Point Lesueur. All animals observed in a spotlight beam were recorded on either side of the vehicle and observations were pooled for each transect. Distances from the observer to individual animals were estimated with the aid of reflective distance markers placed at 25 m intervals perpendicular to the transects, after Le Mar *et al*. [30]. Due to the limited number of possums observed in each survey, observations were pooled across replicates (*n* = 3) within each season for analysis. Detailed methods for marsupial herbivore monitoring surveys on Maria Island are outlined in a previous paper by Ingram [23].

Possum abundance in the grasslands was estimated using the conventional distance sampling analysis engine in DISTANCE statistical software v. 6.2 (http://distancesampling.org/Distance) with a half-normal key function fitted [31] to estimate detection probability. Observations in each survey period were resampled by replacement to estimate variance using non-parametric bootstrap for each replicate transect (× 400 iterations) until the sample size (number of transects) equalled the original survey effort [32]. Final estimates were generated for possum abundances (in open grasslands) in winter and autumn, and mean densities were calculated across both seasons each year (electronic supplementary material, figure S1).

### (d) Island-wide patterns of abundance for possums and devils

We deployed approximately 70 cameras (Reconyx PC600 and PC800, Wisconsin, USA) in summer and in winter on Maria Island, and 50 cameras in winter only at the Rheban control site, from 2013 to 2017 (figure 1). Total deployments were 732 cameras and 20 496 camera-nights. Cameras were spaced at least 500 m apart and angled downwards to capture the area directly beneath a lure station. The lure station included an olfactory lure (PVC canister filled with a mixture of rolled oats, peanut butter, sardines, dried liver, tuna oil, walnut oil and truffle oil) and a visual lure (a blank white CD), both hung from a branch approximately 1.5 m off the ground.

The cameras were programmed to record three consecutive images each time the sensor was triggered, with a 1 s delay between images within sets and no delay between sets. Cameras using HIGH default settings recorded 3.1-megapixel colour images during the day under ambient light and monochromatic images at night under an infrared flash. All images were time and date stamped. Cameras were distributed among grassland, dry and wet forest locations in proportion to land area, with more cameras in dry forest. No cameras were deployed in grassland at the control site due to the risk of camera theft.

As it is difficult to identify individual possums and devils, we derived detectability-corrected estimates of abundance for both species using N-mixture modelling [33], an extension of occupancy modelling [34] that includes detection probability and abundance of species from replicated detection histories (i.e. count data) that often exceed one [33]. To create a detection history for possums and devils, we divided each 28-day survey into four 7-day periods and recorded the number of independent detections (counts) for each species in each period. A detection was defined as independent if separated by 30 min from the previous detection at that site [35]. We treated the estimates from the N-mixture models as detectability-corrected indices of abundance that enabled us to compare trends in abundance between Maria Island and the adjacent control site.

The count data for possums and devils were tested using Poisson or zero-inflated Poisson distributions by creating an intercept-only N-mixture model. The distribution with the lowest Akaike's information criterion (AIC) value was used for further analysis [36]. Negative binomial distributions were not used as they are known to produce unrealistic estimates of abundance [37].

First, we tested our expectation that increasing devil abundance would result in decreasing possum abundance on Maria Island compared with no change in either devil or possum at the control site. We modelled parameters that may influence the detection and abundance of possums and devils on Maria Island and the control site. For the detection component, we tested two parameters: ‘lure age’ that increased from 1 to 4 from the first to the fourth period, and ‘season’ that was either summer or winter. In the possum detection models, we also included ‘devil detections’ per camera, because devil activity at a camera could influence detectability of possums. To estimate temporal changes in the relative abundance of each species, we used the parameters of ‘location’ (Maria Island and control) and ‘year’ (as a proxy for devil abundance, which increased in each successive year of data [21]. Simple additive and interactive models (year × location) were constructed with polynomial terms of ‘year’ and ‘lure age’ to allow for nonlinear effects.

Second, we explored how an increasing devil population influenced possum abundance within the different habitat types (grassland, dry, and wet forest) on Maria Island. The control site was removed as the first analysis indicated no influence of devils on possum abundance. We used multi-model inference and an information-theoretic framework with AIC to rank models and defined the most influential models to be within less than 2 AIC [38]. Top models were tested for fit using the parametric bootstrap function within the ‘unmarked’ package [39].

### (e) Island-wide temporal activity of possums

Temporal activity profiles of possums were created using the time stamp recorded on the camera images. All images of possums from Maria Island were pooled for 2013/2014 (*n* = 1401) and for 2016/2017 (*n* = 1009) to represent periods when the devil population on Maria Island was ‘low’ (*n* ∼ 29 devils) versus ‘high’ (*n* ∼ 100 devils). Non-parametric kernel density curves were fitted to each year using default smoothing parameters to characterize the probability density distribution for possum activity in each period (R package *overlap* v. 0.3.2). The coefficient of overlap, Δ, was calculated as a measure between 0 (no overlap) to 1 (complete overlap) of temporal overlap between estimated distributions for the two time periods, with 95% bootstrap confidence intervals [40]. A non-parametric Watson–Wheeler test was used to test for homogeneity between the two activity periods at each site using the R package *circular* v. 0.4-93 [41]. All analyses were performed on the R v. 3.5.3 statistical computing platform [42].

## 3. Results

### (a) Survival of individual possums in relation to habitat

Home-range maps (*n* = 18) show that possums around Point Lesueur lived either entirely in open grassland or used a mixture of grassland and dry forest (figure 2; electronic supplementary material, table S1). Possums with HRs exclusively in grassland denned in a variety of structures including low shrubs, wombat burrows and rock-piles; those with access to forest denned in tree hollows exclusively (electronic supplementary material, table S1). Fifty per cent of all possums fitted with GPS collars were documented to be killed by devils. Evidence of death by devil was either direct observation of killing when possums were being radio-tracked or the researcher was in the vicinity, or clear signs at the kill (e.g. blood and fur on the ground and the entire carcass, including bones, consumed). We can exclude wedge-tailed eagles and feral cats as cause of death because adult possums (2.5–4 kg) are nocturnal (excludes diurnal raptors) and are too large for feral cats (2–4 kg).

**Figure 3.**
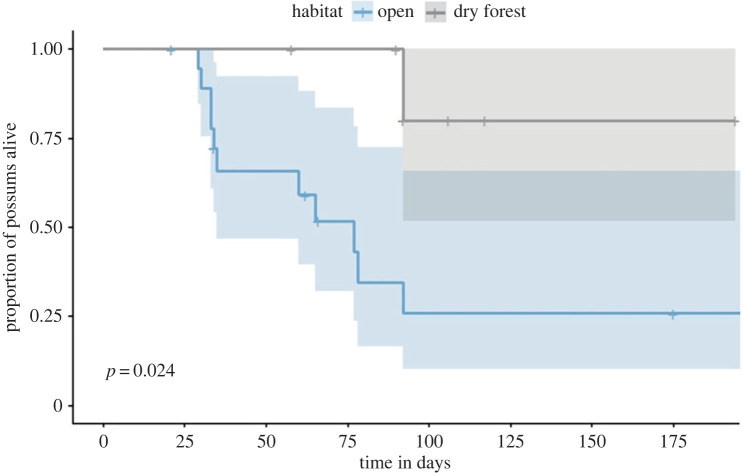
Survival of 18 (12M; 6F) possums fitted with GPS collars from February 2014 until June 2015 on Maria Island at Point Lesueur (PL). Survival of possums was determined as the proportion of possums that used open grassland and dry forest habitats and were not killed by devils (electronic supplementary material, appendix S1: table S1) using the Kaplan–Meier estimate (*p* = 0.024).

Survival was lower for individuals (after approximately 25 days) whose HRs were exclusively in grassland than for those with HRs that overlapped forest and who denned in trees (figure 3). Of the collared possums that survived the study period (*n* *=* *5*), all but one denned in tree hollows (electronic supplementary material, table S1). The exception was a possum who denned in a deep crevice in a rock complex. We were unable to locate this possum after removing its GPS collar, despite extensive camera surveys around the den site, suggesting it also died.

### (b) Change in density of possums in open grassland

**Figure 4.**
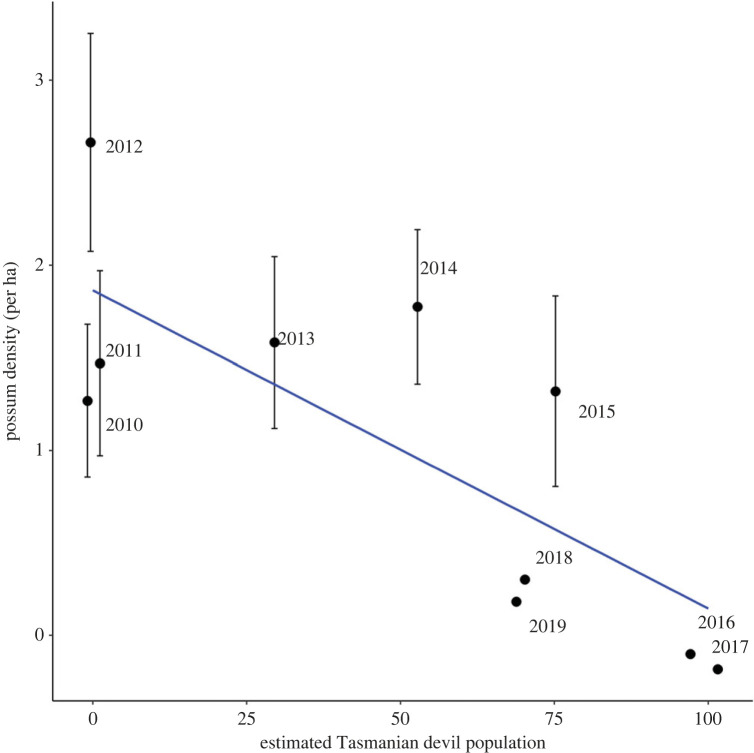
Density estimates of possums in open grassland (approx. 1000 ha) habitats on Maria Island from 2010 to 2018 scaled against population size of devils. Increasing devil density is related to years since their introduction, starting with the introduction of 15 devils in November 2012. Possum population estimates are derived from spotlight survey counts using distance sampling methods for the western part of the island only. The absence of s.e. bars on density estimates reflects none or low counts for those years which could not be estimated using DISTANCE software. Devil populations were derived from regular monitoring by the Save the Tasmanian Devil Program (DPIPWE 2019, unpublished data).

In the 3 years preceding the introduction of devils in late 2012, possum densities in grasslands on Maria Island ranged from 1 to 2.5 individuals per hectare. By 2016 and 2017, when devils had reached their estimated carrying capacity, the density of possums was estimated at zero in grasslands. A small spike in possum density in 2018 coincided with the removal of 30 devils, but density fell back to zero the following year (figure 4).

### (c) Island-wide patterns of abundance and temporal activity of possums

**Figure 5.**
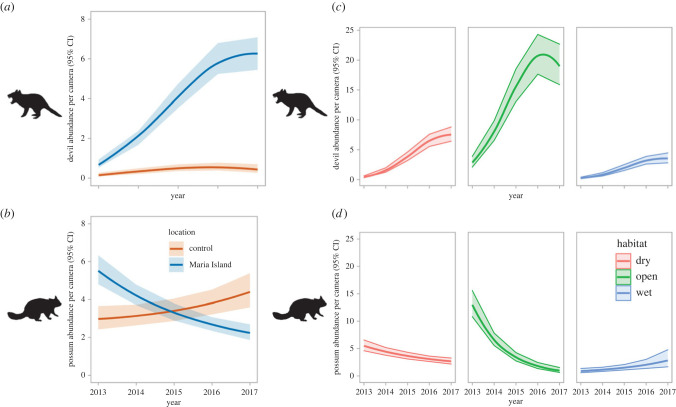
(*a*,*b*) Predicted abundance of (*a*) devils and (*b*) possums on Maria Island and the Rheban control site from 2013 to 2017. (*c*,*d*) Predicted abundance of (*c*) possums and (*d*) devils within the three different habitat types (open grasslands, dry forest, wet forest) on Maria Island throughout the same period (electronic supplementary material, appendix S1, tables S2 and S3).

Before devils became abundant on Maria Island (by 2016–2017), possums were in high abundance across the island (figure 5*b*). Possum abundance was highest in grassland (approx. 15 per camera), followed by dry forest (approx. 5 per camera), and then wet forest (approx. 2 per camera; figure 5*d*). Over the 5 years following the introduction of devils in late 2012, the abundance of possums on Maria Island declined strongly in grassland, less strongly in dry forests, and showed little change in wet forest (figure 5*d*). Possum abundance fell by more than 50% across Maria Island as a whole (all camera sites combined; figure 5*b*) but remained approximately constant at the Rheban control site where the devil population remained low and stable. The ‘year by location’ interaction term had a relative importance (RI) of 0.85 (sum of AIC weights across all models) and was present in the top-ranked N-mixture models describing variation in both devil and possum abundance (electronic supplementary material, tables S2 and S3).

Detection probability for possums and devils on any night was reduced by lure age, and changed with season for devils, being higher in winter than summer. Season had little effect on detection probability of possums. There was a small positive effect of nightly devil detections at camera stations on the detection probability of possums. All detection parameters had a RI of 1.0. (electronic supplementary material, tables S2 and S3).

**Figure 6.**
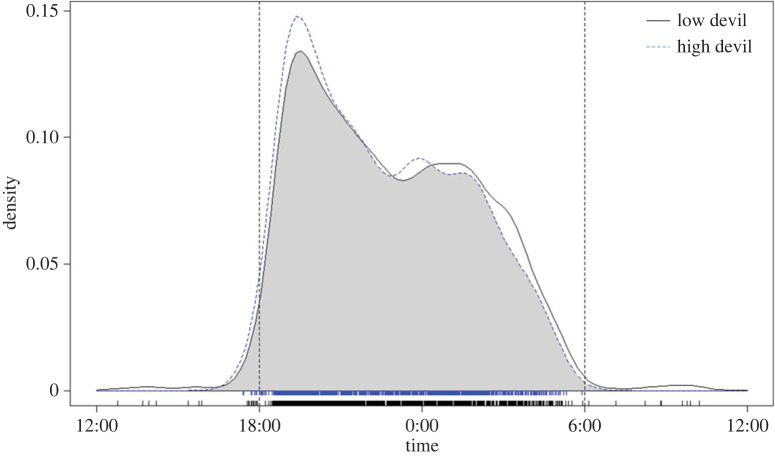
KDEs of brush-tailed possum temporal activity on Maria Island comparing activity in years when the devil population was ‘low’ (2013–2014) and ‘high’ (2016–2017) on Maria Island only. A non-parametric Watson–Wheeler test was used to test these curves for homogeneity.

Possums did not alter their diel activity patterns on Maria Island in response to the increasing devil population (*W* = 1.3758, d.f. = 2, *p*-value = 0.5026; figure 6).

## 4. Discussion

The assisted colonization of Tasmanian devils on Maria Island, intended as a conservation action for the devil, provided an exceptional opportunity to measure the ecological effects of an introduced native top predator. We show that the rapid increase of the devil population caused a contraction in habitat breadth and decline in abundance of common brush-tailed possums. Possums had lived on Maria Island since they were first introduced in the 1950s. In the absence of predation, this typically arboreal folivore expanded its habitat breadth to include open grasslands and became omnivorous by preying on seasonally available short-tailed shearwater (*Puffinus tenuirostris*) adults and chicks in breeding colonies [21]. The introduction and rapid population increase of devils dramatically reduced the survival and abundance of possums, especially in grassland. Possums rely on short escape distances to trees to avoid predators when they come to the ground [43] so are vulnerable in open habitats. Possums in grassland were also vulnerable in their dens, which were mainly burrows dug by wombats and which devils were able to enter. Individual possums whose HRs were completely within grassland, and who denned at ground level, were most likely to be killed. Possums also declined in dry forest, which has an open understorey under widely spaced mature eucalypt trees, but maintained their former abundance in wet forest, where understorey vegetation is denser and more complex, and trees more closely spaced.

Within 5 years of the devil introduction, surviving possums were restricted to forest habitats and to using arboreal tree hollows as den sites. The rapid, island-wide reduction in the population size of possums suggests that the direct effects of predation by devils were the primary cause. This interpretation is supported by four pieces of evidence. First, 50% of the GPS-collared possums in our study, as well as non-collared possums in the shearwater colonies on Maria Island [21], were killed by devils in grassland habitats. Similarly, restoration of top predators to their historic ranges in the USA, Europe and Africa resulted in initial high mortality and swift decreases in populations of native ungulates [4,44,45].

Second, the reduction of possum abundance in grasslands was not matched by corresponding increases in forest habitats, suggesting that devils reduced the overall abundance of possums rather than driving a retreat of possums from grasslands into forested habitats. Likewise, it is possible that the decline in possum abundance within dry but not in wet forests could be attributed to increased mortality. Possums frequently move to the ground to forage, and escape distances to trees would be greater in dry forests than in wet forests.

Third, possums presence comprised 29% of the diet of devils on Maria Island from 2012–2014 [46] compared to 2% approximately 3 years later [47]. This contrast in dietary composition of devils suggests that possums were abundant and easy prey for devils on Maria Island during the period of devil population growth.

Fourth, devil activity had a small but positive effect on the probability of detecting a possum on cameras. This suggests that possums did not greatly alter their behaviour to avoid encounters with devils, possibly reflecting naivety of possums to devils during the initial years of rapid devil population growth. During this period, possums were seen to be ambushed by devils at the lure station at a camera site (V.P.S., J.I., C.N.J. & M.E.J. 2019, personal observation). Conversely, there is evidence that possums on Maria Island did develop risk-sensitive foraging behaviours following introduction of devils: experiments with artificial food trays showed that possums had reduced their foraging distance from trees when devil abundance was high [48]. The results of our study suggest that this change in behaviour was insufficient to counter the large increase in possum mortality in the years immediately after devil introduction and their rapid population growth. In other studies, reintroduction of top predators into ecosystems from which they have been extirpated has resulted in rapid recovery of antipredator behaviours in response to a restored landscape of fear [44,49,50]. Behavioural plasticity in response to changing predator abundance is documented in possums elsewhere in Australia and in other opportunistic omnivore species in the United States and Africa. Possums on mainland Australia are sensitive to the level of predation risk, spending more time foraging on the ground in the absence of predators [51], but they also demonstrate weaker antipredator behaviours than shown in this study [52]. In the USA and Africa, racoons (*Procyon lotor*), opossums (*Didelphis virginiana*) and olive baboons (*Papio anubis*) have swiftly responded to the removal of top predators by increasing their habitat breadth and abundance [53–55].

## 5. Conclusion

This study provides a rare demonstration of the impacts from the assisted colonization of a native top predator on both the abundance and habitat breadth of a widespread prey species. Our results suggest that restoration of native top predators in terrestrial ecosystems may provide an important management tool to assist in limiting the abundance and distribution of overabundant prey species. With a growing interest in restoring top predators globally, further work should also explore impacts on invasive species [56] that are contributing to a loss in diversity—one of the main drivers of the global extinction crisis [57].

## Data Availability

Data can be accessed via the Dryad Digital Repository [58]. Additional data are provided in the electronic supplementary material [59].
